# Overexpression of squamous cell carcinoma antigen variants in hepatocellular carcinoma

**DOI:** 10.1038/sj.bjc.6601543

**Published:** 2004-02-17

**Authors:** P Pontisso, F Calabrese, L Benvegnù, M Lise, C Belluco, M G Ruvoletto, S De Falco, M Marino, M Valente, D Nitti, A Gatta, G Fassina

**Affiliations:** 1 Department of Clinical and Experimental Medicine, Via Giustiniani, 2 35123, Padova, Italy; 2 Department of Pathology, University of Padova, via Gabelli 61, 35121, Padova, Italy; 3 Department of Oncological and Surgical Sciences, University of Padova, via Giustiniani, 2 35123, Padova, Italy; 4 CNR Napoli, via Castellino 111, 80100 Napoli, Italy; 5 Xeptagen S.p.A, via Olivetti, 1, 80078 Pozzuoli (NA), Italy

**Keywords:** serpin variants, HCC, proliferative activity, molecular biology

## Abstract

Pathogenetic mechanisms of hepatocellular carcinoma (HCC) are still unclear and new tools for diagnostic and therapeutic purposes are ongoing. We have assessed whether squamous cell carcinoma antigen (SCCA), a serpin overexpressed in neoplastic cells of epithelial origin, is also expressed in liver cancer. Squamous cell carcinoma antigen was evaluated by immunohistochemistry in 65 HCCs of different aetiology and in 20 normal livers. Proliferative activity was assessed using MIB-1 antibody. In 18 surgical samples, tumour and nontumour liver tissue was available for SCCA cDNA amplification and sequencing. Squamous cell carcinoma antigen was detected in 55 out of 65 (85%) tumour specimens, but in none of the 20 controls. In the majority of the cases, the positive signal was found in the cytoplasm of more than 50% of the hepatocytes. Low or undetectable SCCA (score⩽1) was associated to lower MIB-1 labelling index, compared to cases with SCCA score ⩾2 (mean±s.d.: 2%±2.4 *vs* 7.5%±10.3, *P*<0.05). Squamous cell carcinoma antigen mRNA could be directly sequenced in 14 out of 18 liver tumours but in none of the corresponding nontumour samples. From sequence alignment, a novel SCCA1 variant (G_351_ to A) was identified in five cases, while SCCA1 was revealed in six cases and SCCA2 in three cases. In conclusion, SCCA variants are overexpressed in HCC, independently of tumour aetiology. A novel SCCA1 variant has been identified in one third of liver tumours.

Hepatocellular carcinoma (HCC) is one of the most important sanitary problems over the world for its high prevalence and for its poor prognosis. More than 250 000 new cases per year are diagnosed and mean 5-year survival is lower than 5% (El-Serag and Mason, 1999). Several factors have been involved in the development of liver cancer, some of them differing in various geographic areas and explaining, at least in part, distinct geographical incidence (Bruix *et al*, 2001). The most powerful risk factor is the presence of liver cirrhosis. Among cirrhotic Caucasian patients, chronic viral infection by HBV and HCV are the most biologically relevant causes leading to this clinical condition (Benvegnù *et al*, 1994). However, pathogenetic mechanisms of neoplastic transformation are still unclear; several growth factors and tumour suppressor genes (Collier *et al*, 1993; Naka *et al*, 1998; Abou-Shady *et al*, 1999) have been involved but insufficient data have been generated to date. cDNA microarray analyses for gene expression profiling and potential identification of target genes for diagnostic or therapeutic purposes are ongoing (Shirota *et al*, 2001; Takeo *et al*, 2001; Xu *et al*, 2001).

Squamous cell carcinoma antigen is a serine protease inhibitor physiologically found in the spinous and granular layers of normal squamous epithelium, but typically expressed by neoplastic cells of epithelial origin (Kato, 1996). Recent studies indicate that both SCCA1 and SCCA2, the two isoforms so far identified (Schneider *et al*, 1995), protect neoplastic cells from apoptotic death induced by several kinds of stimuli, and *in vivo* experiments demonstrate that SCCA1 can promote tumour growth (Suminami *et al*, 2000, 2001). Little is known on the behaviour of this serpin in different clinical settings; in particular, no information is available on its expression in hepatocarcinoma. In the present study, we analysed the expression of SCCA in a large series of HCCs of different aetiology, using a novel antibody raised against SCCA variants and cellular transcripts were then characterised by direct sequencing.

## MATERIALS AND METHODS

### Patients

The 65 HCC patients included in the study had a median age of 65 years (range 23–76 years), with a male/female ratio of 2:1. In total, 37 patients were anti-HCV positive, eight were HBsAg positive, six were coinfected by HBV and HCV, eight admitted alcohol abuse, while for the remaining six patients no risk factors were identified. Liver function tests and AFP were available in all cases, as required for clinical management of the patients. Liver specimens, achieved for diagnostic purposes, were obtained by ultrasound-guided fine-needle aspiration of hepatic nodules in 47 patients with cirrhosis, while in the remaining 18 patients, including 12 cases with cirrhosis and six without cirrhosis, specimens of tumour and nontumour liver samples were collected at the time of surgical resection. Formalin-fixed and paraffin-embedded sections were available in all cases, while part of the surgical samples was snap frozen in liquid nitrogen and stored at −80°C for further analysis. Control liver biopsies were obtained from 20 patients who underwent cholecystectomy or liver biopsy for the staging of mediastinal or laterocervical Hodgkin's disease (Calabrese *et al*, 2000).

### Histological evaluation

Paraffin sections were analysed for the presence of SCCA and regeneration activity by immunohistochemistry. For SCCA detection, a novel polyclonal rabbit antibody (Hepa-Ab, Xeptagen, Italy) raised against recombinant SCCA1 and affinity purified on a Sepharose-SCCA1 column was used. Epitope mapping studies with SCCA1 fragments obtained by SCCA1 enzymatic digestion or by chemical synthesis indicated that the affinity-purified polyclonal antibody recognises several epitopes located in the N-terminal, C-terminal as well as in the central portion of SCCA1, as determined by enzyme-linked immunosorbent assay (ELISA) and Western blot. Hepa-Ab was used at a concentration of 4 *μ*g ml^−1^, while anti-Ki67 (MIB-1, Immunotech, Marseille, France) monoclonal antibody was used at 1 : 50 dilution. Sections were incubated with primary antibodies for 30 min, after blocking endogenous peroxidase activity with 3% hydrogen peroxide, heating the slides in 10 mM sodium citrate in a microwave oven and blocking nonspecific protein binding in normal goat serum. Biotinylated goat anti-rabbit or horse anti-mouse (Dako, Copenhagen, Denmark) was then added for 30 min. Samples were incubated with avidin–biotin–peroxidase and stained with a mixture of 3,3′-diamino-benzidine tetrahydrochloride (Dako) and hydrogen peroxide. For all experiments of immunohistochemistry, as negative control, sections were incubated with the omission of primary antibody, substituted by diluent or by the appropriate nonimmune IgG in each case. Antibody specificity was confirmed using human skin specimens for SCCA and colonic cancer specimens for Ki67, as positive controls.

The percentage of stained cells in each specimen was scored on a scale of 0–3, in which 0 denoted negative staining, score 1 positivity in 1–30% of hepatocytes, score 2 positivity in 31–50% and score 3 in more than 50%. The distribution of immunoreactivity was noted and classified as diffuse, clustered or scattered. In all cases, SCCA semiquantitative immunoreactivity was independently evaluated by two pathologists, experts in the field. Intra and interobserver differences were less than 5% and discordant cases were re-evaluated simultaneously by two observers.

For tumour liver specimens obtained by resection, MIB-1 immunostaining was randomly evaluated counting at least 1000 nuclei, while in liver samples obtained by needle aspiration all the cells were counted (maximum 500 cells). The percentage of positive nuclei was expressed as MIB-1 labelling index (MIB-1-LI).

In all immunohistochemical analysis, necrotic areas and edges of tissue sections were not included in the counting to avoid possible immunohistochemical false positivity.

### Virological assessment

Anti-HCV antibody positivity was determined by commercially available ELISA and confirmed by recombinant immunoblot assay version 2 or 3 (Ortho Diagnostics, Raritan, NJ, USA). HBsAg, HBeAg and the presence of serum anti-HBc, anti-HBe and anti-HBs antibodies were evaluated by ELISA using commercially available kits (Abbott Diagnostics, North Chicago, IL, USA). HCV genotype was determined by the InnoLipa test (Innogenetics, Gent, Belgium) after reverse transcription (RT)–PCR amplification. In order to detect possible occult HBV infection, aliquots of liver HBV DNA were assessed in 17 out of 18 HBsAg-negative surgical samples by nested PCR using primers derived from the core and the S regions of viral DNA (Cacciola *et al*, 1999).

### Characterisation of SCCA transcripts

cDNAs of the SCCA-related variants were obtained by RT–heminested PCR starting from total RNA extracted by single-step guanidinium method (Chomczynsky and Sacchi, 1987) from surgically obtained frozen tumour and nontumour liver samples. Samples containing 1 *μ*g RNA were treated with 1 U *μ*l^−1^ of DNAse I in reaction buffer (200 mM Tris-HCl (pH 8.4), 20 mM MgCl_2_, 500 mM HCl), followed by the addition of 25 mM EDTA at 65°C for 10 min to block the reaction. To generate cDNAs of SCCA variants, RT and amplification were carried out using primers located in conserved regions of SCCA1 and SCCA2. Briefly, RT was carried out using 2 pmol of the antisense primer at position 1416–1386 of SCCA sequence (Suminami *et al*, 1991) and 1 *μ*l of reverse transcriptase (Superscript II, GIBCO BRL). For the first step of cDNA amplification, 5 U ml^−1^ of *Taq* polymerase (Experteam, Italy) and the following primers were used: sense (CAT GAA TTC ACT CAG TGA AGC CAA C), antisense (GCA ATC AGT TTA CCA GAA CAT CTG CAG). After 40 cycles at 94°C for 1 min, 60°C for 30 s, 72°C for 1 min, heminested PCR was carried out for additional 40 cycles in the same conditions using the sense primer described above and the antisense primer (GAC TGA ATT CAA ATC CAC TGA TGC). Actin mRNA was used as control of extracted RNA and RT–PCR was assessed using the following primers: sense (GTG GGG CGC CCC AGG CAC CA), antisense (CTC CTT AAT GTC ACG CAC GAT TTC). cDNA amplification was carried out for 35 cycles at 94°C for 1 min, 55°C for 1 min and 72°C for 2 min.

Samples positive for the expected 945 bp band obtained with the SCCA-derived primers were directly sequenced in both strands using an ABI PRISM BigDay terminator ready reaction kit following the manufacturer's instructions (Perkin Elmer Cetus, Emeryville, CA, USA). Electrophoresis of the sequencing products was performed by an ABI 377 automated DNA sequencer (Perkin Elmer Cetus) according to the manufacturer.

### Statistical analysis

The Kruskal–Wallis ANOVA median test, Spearman rank correlation, *χ*^2^ and Fisher exact test were used for the analysis of the results.

## RESULTS

### Immunohistochemistry

**Figure 1:**
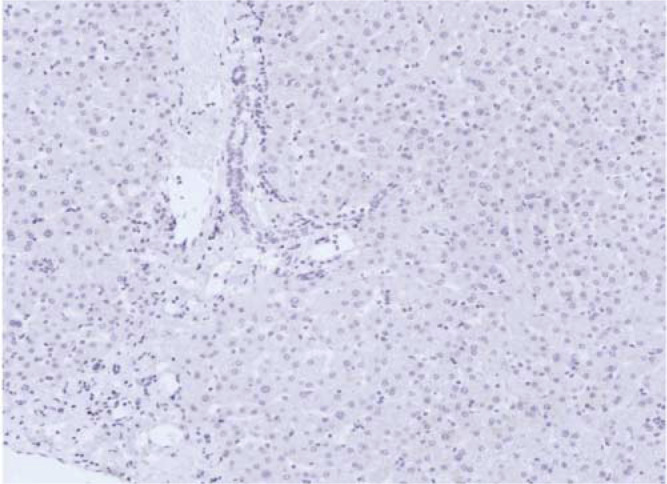
Negative liver staining for SCCA variants by immunohistochemistry in normal human liver (staging liver biopsy from a patient with mediastinal Hodgkin's disease). Original magnification: × 20.

**Figure 2:**
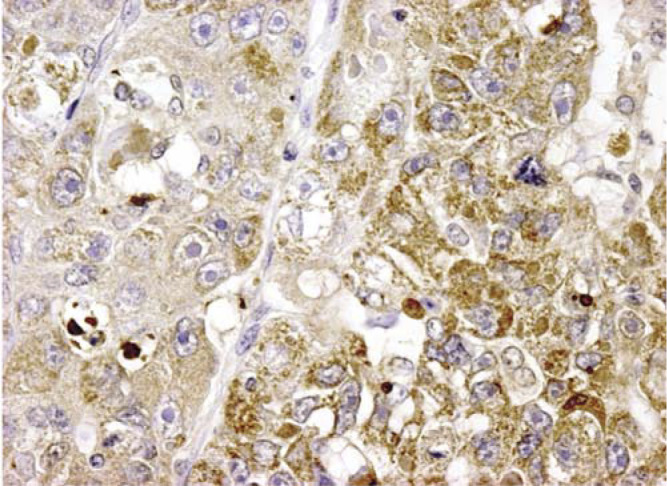
Immunohistochemistry for SCCA variants (score 3) in poorly differentiated HCC (giant cell form): most of the tumour cells show typical clustered, coarse, brown granules. Original magnification: × 572.

Squamous cell carcinoma antigen was detected by immunohistochemistry in 55 out of 65 (85%) tumour specimens (18 out of 18 surgical specimens and 37 out of 47 fine-needle liver biopsies), but in none of the 20 surgically obtained normal human livers (Figure 1

). In the majority of the cases, the positive signal was clearly detectable with score 1 in 15% of the cases, score 2 in 29% and score 3 in the remaining 56% of the patients. The same extent of immunoreactivity was observed in liver tumours expressing SCCA1, SCCA2 or SCCA-PD variants as detected by direct sequencing (see below). In all cases, the serpin was detected in the cell cytoplasm, with a prevalent diffuse pattern. In poorly differentiated tumours, a typical clustered, coarse pattern was observed (Figure 2

). No correlation with the diameter, histotype or grading of the tumour was observed. When surgical liver specimens were considered, patients with cirrhosis showed a higher score, compared to cases without cirrhosis (mean score±s.d.: 3±0.7 *vs* 2±0.4, *P*<0.05).

To correlate the presence of SCCA with liver regeneration activity, consecutive slices of each tumour were stained with the Ki-67 equivalent, MIB-1 antibody, which identifies cycling cells in formalin-fixed tissue samples (Cattoretti *et al*, 1992).

**Figure 3:**
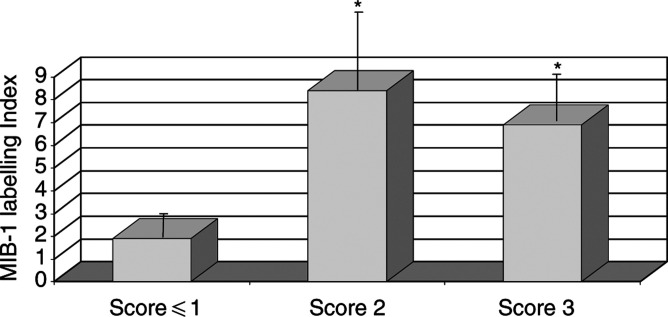
Proliferative activity expressed as MIB-1-labelling Index in HCCs with different SCCA scores. Results are expressed as percent mean+s.e. ^*^P<0.05 *vs* score ⩽1 group, Student's paired *t*-test.

Patients with SCCA score ⩽1 showed values of MIB-1-LI significantly lower than those obtained in patients with serpin score ⩾2 (mean+s.d.: 2%±2.4 ([range 0.1−6.3%)] *vs* 7.5%±10.3 ([range 0.2−48%)], *P*<0.05), as shown in Figure 3

.

### Squamous cell carcinoma antigen transcripts analysis

**Figure 4:**
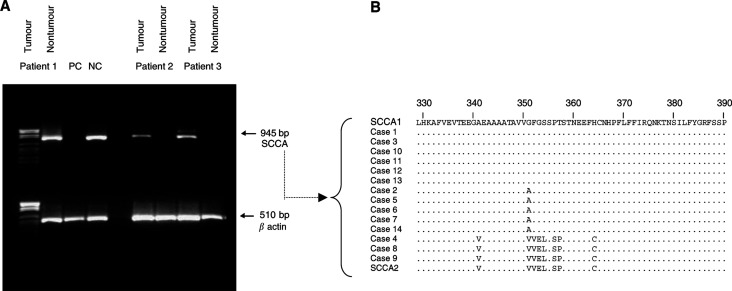
(**A**) Example of PCR amplification of SCCA cDNA by heminested PCR in tumour and nontumour liver tissue, surgically obtained from the same patient. Parallel amplification of *β* actin cDNA is used as control of cellular mRNA. PC=positive control; NC=negative control. (**B)** Sequence alignment of SCCA amino-acid sequences obtained from direct sequencing of cDNA of 14 HCCs. The sequence at the top is SCCA1 and the sequence at the bottom is SCCA2 (Suminami *et al*, 1991). Amino-acid changes differing from SCCA1 reference sequence are reported. Cases 2, 5, 6, 7, 14 show the G_351_ → A mutation (SCCA-PD).

Squamous cell carcinoma antigen mRNAs were detectable by heminested PCR in 14 out of 18 tumour samples, generating a single band at the expected size of 945 bp. None of the corresponding nontumour liver tissues showed detectable levels of the serpin in the same experimental conditions (Figure 4A

). The sensitivity of this molecular approach was lower than that of immunohistochemistry for SCCA, where all tumour samples were positive for the serpin. The nucleotide sequence alignment of the cDNA obtained by direct sequencing from the 14 tumours showed that the major SCCA sequence had complete homology with SCCA1 in six cases and with SCCA2 in three cases. In five additional cases, a novel variant, presenting a G_351_ to A mutation, in the reactive centre loop of the protein, was identified (Figure 4B) and termed SCCA-PD (GenBank accession number: AY190327).

### Correlation with clinical and virological parameters

The immunoreactivity for SCCA variants in liver tumours did not show any relation with clinical and biochemical parameters, including age, sex, transaminase or AFP levels. Aetiology of HCC did not correlate with serpin expression, the mean±s.d. score being 2.1±1.1 in HCV-infected patients, 2.1±1.7 in HBsAg-positive patients, 2.2±0.4 in patients with HBV and HCV coinfection, 2.1±1.6 in patients with alcohol abuse and 2.0±0.5 in cases without overt risk factors. Occult positivity for HBV was detected in five out of 17 surgical samples obtained from HBsAg-negative patients, independently of anti-HBc or anti-HBs positivity. In HCV-positive patients, no correlation with the infecting genotype was observed.

## DISCUSSION

In view of its prevalence and poor prognosis, HCC is a main concern. This is the first report of an high expression of SCCA in human liver cancer, detected in all surgical tumours and in 79% of the samples obtained by fine-needle aspiration, confirming a lower sensitivity of the single fine-needle procedure (Borzio *et al*, 1994). The majority of HCCs displayed the serpin at cytoplasmic level, while its reactivity was not detectable in normal human livers. The presence and extent of immunoreactivity, as detected using a novel anti-SCCA antibody, was not correlated with aetiologic risk factors, suggesting that overexpression of this protein is involved in pathologic stages, beyond promotion of cell transformation.

The role of serpins in neoplastic cells has been focused in several studies and recent reports indicate that SCCA expression makes cancer cells resistant to several killing mechanisms by inhibition of apoptosis, involving caspase-3 activity and/or upstream proteases (Suminami *et al*, 2000). So far, two isoforms of SCCA (SCCA1 and SCCA2) deriving from two highly homologous tandemly arrayed genes and their promoter regions have been identified on chromosome 18q21.3 (Schneider *et al*, 1995; Sakaguchi *et al*, 1999; Hamada *et al*, 2001). In this study, direct sequencing was used to characterise the expression of the major species of SCCA variants in individual tumours and a new variant has been identified in about one third of the cases, which is 99% identical to SCCA1, but presents a G_351_ to A mutation in the reactive centre of the protein. Since the mechanism of protease inhibition by serpins involves a profound change in conformation, initiated by interaction of the protease with the reactive centre of the serpin (Huntington *et al*, 2000), the specific amino-acid change detected in the reactive centre of SCCA-PD might confer a different biological behaviour to the serpin and enzymatic activity of this new variant is currently under investigation. Mutations affecting this region may indeed result in inhibition of different classes of proteinases, as shown for SCCA1 and SCCA2 (Kato, 1996) or in a loss or change of function, as described in several human diseases affecting different members of the ovalbumin family of serpins, including emphysema and cirrhosis, haemorragic diseases, thrombosis and familial angioedema (Carrell and Lomas, 2002).

The SCCA-PD variant was detected in one third of the cases and the limited number of patients did not allow any correlation with clinical or morphological parameters. Further studies are required to assess whether tumour behaviour and clinical outcome of patients with HCC are influenced by the extent and/or type of SCCA expression in individual tumours.
