# Overexpression of squamous cell carcinoma antigen variants in hepatocellular carcinoma

**DOI:** 10.1038/sj.bjc.6602326

**Published:** 2004-12-21

**Authors:** P Pontisso, F Calabrese, L Benvegnù, M Lise, C Belluco, M G Ruvoletto, S De Falco, M Marino, M Valente, D Nitti, A Gatta, G Fassina

**Correction to:** *British Journal of Cancer* (2004) **90**, 833–837. doi:10.1038/sj.bjc.6601543

Due to an author error, Dr S de Falco was incorrectly included as an author without his agreement. His name should be deleted and the corrected author list is as follows:

P Pontisso, F Calabrese, L Benvegnu, C Belluco, MG Ruvoletto, M Marino, M Valente, D Nitti, A Gatta and G Fassina

**Editors Note:**

It is the responsibility of the corresponding author to ensure that each co-author on a joint paper has fully agreed to authorship before signing the copyright agreement, and publication.

